# dsRNA-Dependent Protein Kinase PKR and its Role in Stress, Signaling and HCV Infection

**DOI:** 10.3390/v4112598

**Published:** 2012-10-29

**Authors:** Stéphanie Dabo, Eliane F. Meurs

**Affiliations:** Unit Hepacivirus and Innate Immunity, Department Virology, Institut Pasteur, 28 rue du Dr Roux, 75724 Paris Cedex 15, France; Email: sdabo@pasteur.fr

**Keywords:** PKR, dsRNA, IFN, eIF2α, PRR, HCV, MAVS, ISG15, RIG-I

## Abstract

The double-stranded RNA-dependent protein kinase PKR plays multiple roles in cells, in response to different stress situations. As a member of the interferon (IFN)‑Stimulated Genes, PKR was initially recognized as an actor in the antiviral action of IFN, due to its ability to control translation, through phosphorylation, of the alpha subunit of eukaryotic initiation factor 2 (eIF2α). As such, PKR participates in the generation of stress granules, or autophagy and a number of viruses have designed strategies to inhibit its action. However, PKR deficient mice resist most viral infections, indicating that PKR may play other roles in the cell other than just acting as an antiviral agent. Indeed, PKR regulates several signaling pathways, either as an adapter protein and/or using its kinase activity. Here we review the role of PKR as an eIF2α kinase, its participation in the regulation of the NF-κB, p38MAPK and insulin pathways, and we focus on its role during infection with the hepatitis C virus (HCV). PKR binds the HCV IRES RNA, cooperates with some functions of the HCV core protein and may represent a target for NS5A or E2. Novel data points out for a role of PKR as a pro-HCV agent, both as an adapter protein and as an eIF2α-kinase, and in cooperation with the di-ubiquitin-like protein ISG15. Developing pharmaceutical inhibitors of PKR may help in resolving some viral infections as well as stress-related damages.

## 1. Structure of PKR

**Figure 1:**
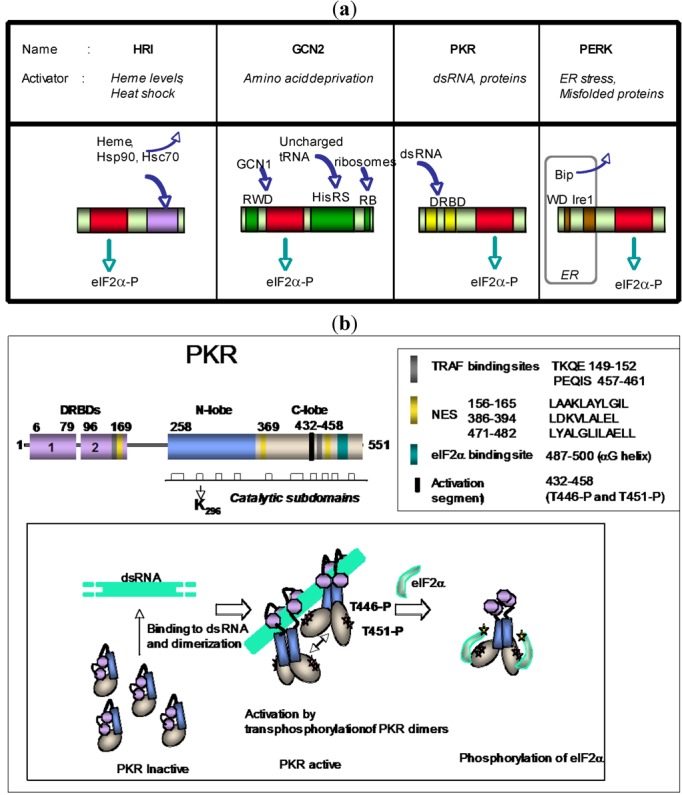
(**a**) The eIF2α-kinase family. Schematic representation of the four eIF2α-kinases: HRI, GCN2, PKR and PERK, showing their catalytic domains (in red) located at the N terminus (HRI, GCN2) or C terminus (PKR and PERK). HRI activates in response to diminution of heme levels or binding to some heat shock proteins such as Hsp90 or Hsc70. GCN2 form an inactive dimer through its region homologous to histidyl-tRNA synthetase (HisRS) and becomes activated, by change of conformation, when this domain binds uncharged tRNA, allowing its N-terminal ring finger and WD repeat domain (RWD) to bind GCN1 required for its function. It contains also a Ribosomal Binding site (RB) at its C terminus. PKR activates through two basic domains or dsRNA binding domains (DRBD) located at its N terminus. PERK is maintained in an inactive state through binding to the chaperone Bip protein (also known as Grp78 or Hsp 70-5) at its luminal N terminus (on WD repeats and Ire1-like domain). Unfolded proteins appearing in the lumen during ER stress attract Bip, which allows PERK homodimerization and activation of its cytosolic kinase domain. (**b**) PKR structure and mode of activation. The two 73 aa dsRNA Binding Domains (DRBD1 and DRBD2), located at the N terminus of PKR (purple) are responsible for the binding of PKR to its regulators. Mutation of the K296 residue (in subdomain II of the 253–525 catalytic domain; white bars) is sufficient to abrogate the catalytic activity of PKR. The sequences of the TRAF binding sites (grey bars) and nuclear export sequences (NES; yellow bars) is as indicated in the upper box. The eIF2α substrate binds in the helical C-lobe of PKR (blue bar) and the activation segment (black) contains the threonines 446 and 451. Lower box: Representation of the activation process of PKR upon binding to dsRNA. After binding of the first DRBD to dsRNA, binding of the second DRBD stabilizes the dsRNA/PKR complex and opens the conformation of PKR. This allows PKR dimerization through the N-lobe of its kinase domain (blue). PKR dimers activate each other by transphosphorylation of their T446 and T551 residues (red stars). The substrate (here eIF2α; green) can then access to its docking site and is positioned correctly within the catalytic domain of PKR where its acceptor site (yellow star) can receive the phosphate from ATP.

PKR is a 551 amino acid protein [1] that can be linked to two different families of proteins: The eIF2α-kinase family and the dsRNA-binding protein family. Through its serine/threonine kinase domain located at its C terminus, PKR belongs to the eIF2α-kinase family, that contains also GCN2 (general control nonrepressed 2), PERK (PKR-like Endoplasmic Reticulum Kinase) and HRI (Heme‑Regulated eIF2 alpha kinase). These are activated in response to amino-acid deprivation (GCN2), overload or to misfolded proteins in the endoplasmic reticulum (ER) (PERK) and diminution of heme levels or heat shock (HRI), respectively [2] (Figure 1a). The N terminal structure of PKR contains two basic, helical domains, with an αβββα topology, referred to as dsRNA Binding Domains (DRBD) that link PKR to the large family of proteins, with one or several dsRNA-binding motifs [3]. Among this family, the TAR RNA Binding Protein (TRBP) and the PKR Activator (PACT) stand out as PKR regulators because, in conditions of stress and even in the absence of dsRNA, they have the possibility to activate (PACT) or inhibit (TRBP) the activity of PKR through interaction between their DRBDs [4]. dsRNA binds to the DRBD of PKR through contact with OH-groups and with phosphates and in a sequence-independent manner. PKR does not interact with dsDNA or RNA/DNA hybrids. Under its inactive state, PKR presents disordered regions, which become ordered when PKR binds RNA [5]. Binding occurs first between the dsRNA and the first DRBD motif, then stabilisation of PKR on dsRNA occurs after binding on the second DRBD (Figure 1b). Binding to dsRNA or PACT triggers a change in the conformation of PKR which allows its dimerization through the N-lobe of its kinase domain in a back-to-back orientation and then access of its substrate-docking site in the helical C-lobe of this domain. Binding of the substrate requires autophosphorylation of T446 in the activation loop of PKR, an event which occurs after dimerization of PKR [6]. Because of the back-to-back dimerization, trans-autophosphorylation is not permitted. Therefore, phosphorylation may occur in cis or from another PKR dimer [7]. This explains why a minimum length of 33 bp dsRNA is required to activate PKR as this length is needed to accommodate the binding of two PKR molecules. Within the activation loop, the segment 448–458 allows the docking of the phosphoacceptor sequence of the substrate. A threonine at position 451 confers PKR its specificity as a serine/threonine kinase. However, some distortion can occur around this location, which allows PKR to display some dual specificity, and to phosphorylate tyrosine residues in some circumstances [7]. Phosphorylation of T446 is a prerequisite for the phosphorylation of T451 [8]. Once the substrate is correctly in place within the catalytic domain of PKR, the kinase mediates the transfer of phosphate from ATP to the acceptor site of the substrate (Figure 1b). Mutation of its residue K296, located in the catalytic subdomain II of PKR abrogates its catalytic activity [9]. PKR contains also TRAF-binding motifs in the second DRBD and at the C terminus [10]. This may allow PKR to bind cellular partners and be engaged in several complexes by protein-protein interactions as developed further below. 

## 2. Cellular Localization of PKR

PKR is an ubiquitous protein, which is usually expressed at a low constitutive level. Its promoter contains a canonical interferon (IFN)-stimulated response element (ISRE), GAAAACGAAACT, and its expression can be induced in response to treatment by type I IFN [11]. PKR localizes predominantly in the cytoplasm and can also be detected in the nucleus. Indeed, PKR contains three nuclear export signals (NES) [12,13] (Figure 1b). In the cytoplasm, PKR is expressed as a somewhat diffuse and punctuate pattern [14]. Its retention to the cytoplasm is favored by the presence of its DRBDs, as shown by using different PKR-GFP constructs [15] or by transfecting different PKR constructs into PKR-deficient MEFs [16]. PKR interacts with ribosomes, through its DRBDs and also through its kinase domain [17]. Accordingly, in the nucleus, PKR is more concentrated in the nucleolus, where ribosomes are assembled and processed while it is diffusely expressed in the nucleoplasm [15]. The nuclear localization of PKR is not affected by IFN treatment [18] but may vary depending on the viral infection. For instance, no effect was noted upon infection with Adenovirus 2 [19], whereas infection with Human Cytomegalovirus (HCMV) infection results in the accumulation of PKR in the nucleus [20]. Importantly, nuclear PKR presents a phosphorylation status different from the cytoplasmic PKR [21]. Accumulation of phosphorylated PKR in the nucleus is considered as a response to cellular stress and it has been linked to some pathologies, such as leukemic development [22,23] and sporadic Alzheimer disease [24,25] or Creutzfeldt-Jakob disease (CJD) [26]. 

## 3. PKR and eIF2α-Kinase Activity

### 3.1. eIF2α-Kinase

**Figure 2:**
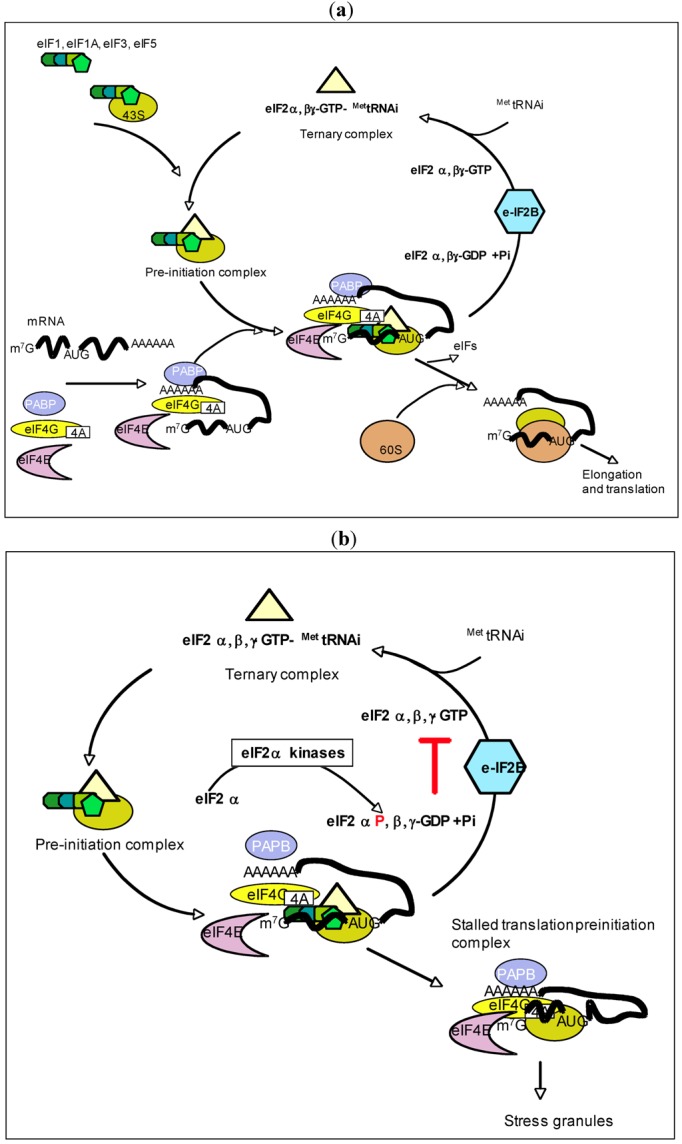
(**a**) eIF2 and initiation of translation. The initiation factors eIF1,eIF1A, eIF3 associate with eIF5 and the ternary complex eIF2α,β,γ-GTP-^Met^tRNA_i_ to form the pre‑initiation complex on the 43S ribosomal subunit. The mRNA associates with the eIF4A/4G/4A complex through its 5' monomethylated guanosine cap structure (abbreviated as m7G) and with the PolyA Binding Protein (PABP) at its polyadenylated 3'end. After association of all the components on the 43S, the preinitiation complex scans the RNA for recognition of the AUG initiation codon. This is followed by binding of ^Met^tRNA_i_, and hydrolysis of GTP in the ternary complex, which is thus released as the binary complex eIF2α,β,γ-GDP together with free phosphate (Pi) while the 60S ribosomal subunit binds to the RNA/43S complex. The eIFs are then released and the process of elongation and translation begins. The binary complex eIF2α,β,γ-GDP associates with the large eIF2B protein complex to be regenerated as ternary complex. It can then associate with a new^ Met^tRNA_i_ and start a novel round of initiation of translation (adapted from [29]). (**b**) eIF2α kinases and control of initiation of translation. Activation of the eIF2α kinases (PKR, PERK, GCN2, HRI) under conditions of stress trigger the phosphorylation of the α subunit of eIF2 in the eIF2α,β,γ-GDP complex. This prevents the replacement of GDP by GTP within the eIF2B complex and inhibition of the initiation process. The translation pre‑initiation complex is then blocked and the different components are used for the formation of stress granules.

The alpha subunit of eukaryotic initiation factor 2, or eIF2α, was identified as a substrate for PKR in the early 80s, following the initial observation that a dsRNA-dependent kinase was involved in the inhibition of translation through eIF2α phosphorylation in rabbit reticulocyte lysates [27] (review [28]). eIF2α is one subunit of the initiation factor eIF2 within the ternary complex eIF2-GTP-^Met^tRNA_i_ that is required, together with other initiation factors, for the correct position of ^Met^tRNA_i_ on the 43S ribosomal subunit. It is released upon hydrolysis of GTP once the 43S ribosomal subunit has been docked to the AUG initiation codon, either after ribosome scanning or direct attachment through Internal Ribosomal Entry Sites (IRES) [29] (Figure 2a). PKR binds on a conserved surface of eIF2α through its domain αG located towards the end of its C-terminus domain. This helps a correct positioning of the Ser51 phosphoacceptor site of eIF2α toward the PKR kinase active site [30] (Figure 1b). Phosphorylation of eIF2α prevents the regeneration of GTP in the ternary complex eIF2‑GTP-tRNA^Met^ and hence halts the initiation of translation (Figure 2b). The ability of human PKR to phosphorylate eIF2α has been demonstrated *in vivo*, first in yeast leading to an expected inhibition of growth [31] and second after stable expression in murine cells [32]. PKR has been rapidly proposed as a powerful antiviral agent through its ability to activate upon appearance or accumulation of viral dsRNAs and to trigger the eIF2α-P mediated inhibition of translation of viral RNAs [28].

### 3.2. eIF2α Phosphorylation and Viral Escape

As eIF2α phosphorylation negatively regulates the two major modes of translation, *i.e.*, involving ribosome scanning or direct attachment to IRES, a number of viruses have evolved with strategies to escape this control. A good example is that of Poxviruses which express PKR inhibitors such as the vaccinia virus K3L protein which presents 33% identity with eIF2-α and competes with its substrate binding site on PKR [33]. In the case of poliovirus, translation is first sensitive to eIF2α phosphorylation but becomes resistant later in infection, because of the ability of the 3C^pr^° protein of this enterovirus to cleave and use the C-terminus of eIF5B. This fragment may behave as the prokaryotic initiation factor IF2 and helps to recruit ^Met^tRNA_i_ or use alternative tRNA initiating at non‑AUG start codons [34]. The second cistron of the picornavirus-like cricket paralysis virus genomic RNA was shown to direct the incorporation of an alanine instead of the canonical methionine to circumvent the block due to eIF2α-P and start translation [35]. Alphaviruses, such as Sindbis virus and Semliki Forest virus can stall the scanning of ribosomes by a hairpin loop RNA structure and use initiation factor eIF2A which can direct binding of the ^Met^tRNA_i_ to 43S ribosomal subunits in a AUG codon-dependent manner [36]. Translation from the IRES of Encephalomyocarditis Virus (EMCV) is inhibited by eIF2α phosphorylation, that of IRES of Hepatitis C virus (HCV) is not [37,38]. In this case, HCV is also able to use eIF2A to favor its translation in an environment rich in phosphorylated eIF2α [39]. *In situ* ations where eIF2α phosphorylation triggers an arrest of translation, the untranslated RNAs are redirected from polyribosomes to stress granules (Figure 2b) [40]. PKR is one among the four eIF2α kinases and each of them can therefore direct the formation of stress granules. A direct link between PKR and stress granules has been recently demonstrated in response to infection by the flavivirus West Nile Virus [41] by comparing the generation of stress granules under viral infection using conveniently mouse embryonic fibroblasts (MEFs) that are deficient for HRI [42], PERK, GCN2 [43] or PKR [44]. Recent results confirmed the participation of PKR in the formation of stress granules in response to dsRNA and infection with HCV [45,46] or influenza [47]. 

### 3.3. eIF2α Phosphorylation and Impact on Cell Metabolism

Some eukaryotic RNAs can benefit or escape from control on translating through eIF2α phosphorylation, whether triggered by PKR or the other eIF2α kinases. While general protein synthesis is inhibited because of eIF2α phosphorylation, the secondary structure of untranslated ORFs in the 5' UTR of some RNAs can escape the translational block and help the cells to recover from stress. For instance, the GCN2-mediated inhibition of translation in yeast upon amino-acid deprivation leads to an increase in the translation of GCN4, which allows this transcription factor to direct novel synthesis of amino-acids [48]. Similarly, the PERK-mediated inhibition of translation upon misloading or overload of proteins in the ER leads to preferential translation of the Activating Transcription Factor 4 (ATF4). This transcription factor then directs transcription of several effectors: CHOP (c/EBP Homologous Protein), which leads to programmed cell death, the ER chaperone BiP (Binding Immunoglobulin Protein), which helps resolving protein aggregation in the ER lumen, and GADD34 (Growth Arrest and DNA Damage Gene) which negatively regulates the stress-induced events by interacting with Protein Phosphatase 1 (PP1) to dephosphorylate eIF2α [43,49]. Plaque amyloid, also referred to as β‑amyloid aggregates, is a major histopathological hallmark of Alzheimer Disease. β-amyloid is formed from the Amyloid Precursor Protein (APP). Here again, extensive secondary structure has been identified in the 5' UTR mRNA of the Beta-site APP-Cleaving Enzyme (BACE-1), which is involved in the formation of β-amyloid. Translation of BACE-1 is increased in the presence of phosphorylated eIF2α, in response to an energy metabolism stress, such as glucose deprivation and PERK activation [50] or oxydative stress (H_2_O_2_) and PACT-mediated PKR activation [51]. It should be noted here that phosphorylation of eIF2α represents one of the mechanisms by which PKR triggers apoptosis (not developed here and reviewed in [28]). PKR-mediated apoptosis is thought to represent a major threat for the development of Alzheimer's disease. In support of this, phosphorylated PKR and its PACT activator have been shown to colocalize in brains from Alzheimer’s disease (AD) [52].

### 3.4. eIF2α Phosphorylation and Autophagy

In response to a stress such as nutrient deprivation or viral infection, cells make use of their eIF2α kinases to inhibit the translation of unnecessary or unwanted protein synthesis. In addition to this, cells are able to activate an autophagy process, which allows either to maintain their homeostasis, through rapid recycling of nutrients, or to destroy incoming unwanted guests, such as bacteria or viruses. The biogenesis of autophagosomes is complex, it starts at the level of intracytoplasmic double-membrane non-degradative vacuoles and involves several Autophagy (Atg)-related proteins, such as Beclin1, the Beclin 1/phosphatidylinositol 3-kinase (PI3K) complex, the Atg5-Atg12-Atg16L multimeric complex and the lipidated form of LC3 (microtubule-associated protein light chain 3) [53]. Demonstration for a role for PKR, GCN2 and eIF2α phosphorylation in the generation of autophagy was provided using yeast and PKR deficient MEFs. The ICP34.5 product of the Herpes Simplex Virus 1 (HSV-1) has been recognized as an antagonist of the PKR-mediated autophagy [54]. ICP34.5 was then shown to act by binding to Beclin 1 [55]. Other viruses such as Human Herpes Virus-8 (HHV-8), Human Immunodeficiency Virus (HIV), influenza A and Human Cytomegalovirus (HCMV) can also counteract autophagy by interacting with Beclin and other crucial Atg proteins (reviewed in [56]). The mechanisms leading to initiation of autophagy are still unclear but several lines of evidence indicate that it may initiate at the level of the endoplasmic reticulum and involves second messengers such as Phosphatidyl inositol phosphates.

## 4. Physiological Role of PKR

### 4.1. *In Vivo* Antiviral Activity

As an IFN-induced, dsRNA-activated protein kinase that can control protein synthesis, PKR is considered as one of the major players in the antiviral action of IFN. Indeed, a number of viral‑encoded products are able to strongly counteract the action of PKR, for instance at the level of its activation or at the site of its catalytic activity [28,57]. Nevertheless, a full appreciation of the *in vivo* antiviral activity of PKR requires analysis of the response of PKR deficient mice to viral infections. Two PKR deficient strains have been generated, one by targeted disruption of exon 2 and 3 (the latter carrying the ATG start) in a 129/EvSv × C57BL/6J background [44] and the other by targeted disruption of exon 12 within the catalytic domain in a 129/terSv × BALB/c background [58]. Analysis of the two strains was at first disappointing as in both cases, mice were viable, fertile, healthy and presented normal anti-viral responses after intravascular inoculation of EMCV [44] or Vaccinia virus [58]. However, a striking finding arose when the PKR-exon 12 targeted deficient mice succumbed to intranasal infection with Vesicular Stomatitis Virus (VSV) and showed increased susceptibility to influenza virus infection [59]. The discrepancy was then found to be related to differences in surrounding genetic backgrounds which may compensate or not for the PKR defect. Indeed, generation of PKR deficient mice in the 129/EvSv mice by successive backcross resulted in higher sensitivity to intranasal infection with VSV [60]. Another example of the regulation of PKR activation by the cellular background comes from the expression of HIV in human cells. Indeed, this virus has evolved to replicate in cells that express cellular factors, which are able to regulate PKR [61]. These factors are TRBP ([4,62] and reviewed in [63]), PACT [4,62] and the adenosine deaminase acting on RNA ADAR1 whose expression is induced by HIV and which binds to PKR and inhibits its activation [64]. The inhibition of PKR activation by ADAR1 has also been demonstrated during VSV [65,66] and Measles virus infections [67,68,69]. Therefore, the importance of PKR as an anti‑pathogenic agent must be considered in each case as a function of the nature of the virus and of its route of infection. Of note, the ADAR1 gene has been recently added [70] to the list of genes encoding important suppressors of type IFN signaling such as the Three Prime Repair Exonuclease 1 (TREX1), Ribonuclease H2 (RNASEH2) and SAM domain and HD domain-containing protein 1 (SAMHD1). Mutations in these genes cause chronic inflammation in the central nervous system, such as in the autoimmune disorder Aicardi-Goutières syndrome [71,72]. 

### 4.2. PKR as a Cognitive Decline Biomarker

Recent data also highlighted difference between PKRwt and PKR deficient mice (in the 129/EvSv background) by showing that PKR could be involved in the control of cognition. This was linked to its binding to pseudoknot structures present at the 5' end of IFNγ mRNA and localized inhibition of the translation of this mRNA. As a result, IFNγ can no longer control the excitability of the neurones through the reduction of the production of acide γ-aminobutyrique (GABA) at the synapses. PKR deficient mice thus are doted with a prolongation of long-term memory [73]. These data are well in line with previous data that identified PKR as a cognitive decline biomarker in patients suffering from Alzheimer disease by showing correlation between cognitive and memory test scores with PKR activation and breakdown of translation [74].

### 4.3. Regulation of Signaling Pathways

As shown in the previous sections, PKR plays a major role in the regulation of some cellular functions through its ability to phosphorylate the initiation factor eIF2α, its major substrate. However, PKR is also involved in the phosphorylation of other substrates and can participate in some signalling pathways, even in the absence of its catalytic activity. Some examples are given below and, when necessary, care has been taken to mention whether these functions may still involve PKR-mediated control of translation through eIF2α or are independent of this. 

#### 4.3.1. PKR and Tumor Suppressors

Cells expressing catalytically inactive mutants of PKR can grow as solid tumors in nude mice, which initially implied that PKR might be a tumor suppressor [75,76]. However, PKR deficient mice did not show any sign of spontaneous tumor. It is now thought that a forced expression of a catalytically inactive PKR mutant may change the conformation of the PKR structure in such a way that it favors the activation of signaling pathways leading to deregulation of cell proliferation. However, PKR can play some role in the control of cell proliferation upon DNA damage or stress, through the tumor suppressor p53. This protein is subjected to a complex regulation involving protein‑protein interaction and phosphorylation. PKR was shown to phosphorylate human p53 on Ser^372^ *in vitro* an *in vivo* and to participate in signaling pathways leading to p53 activation, such as p53-mediated transcriptional transactivation of p21 and mdm2 genes [77,78]. The mechanism underlying this was recently reported to occur after stress-mediated activation of PKR, through PACT, using a sumoylation-dependent mechanism with promotion of p53 phosphorylation and translational activation leading to G(1) arrest [79].

#### 4.3.2. PKR and the NF-κB Pathway

Because of its ability to bind dsRNA, a role for PKR in the process of IFN induction has been considered long before the identification of pathogen recognition receptors such as Toll Like Receptor 3 (TLR3) or the two RNA helicases, Retinoic Inducible Gene 1(RIG-I) and Melanoma Differentiation-Associated protein 5 (MDA5) [80]. In regards with IFN induction, no difference could be detected between PKRwt and PKR deficient mice, either after injection of the synthetic dsRNA, polyinosinic acid: Polycytidylic acid (polyrI-polyrC) or infection with Newcastle Disease Virus (NDV) as shown by induction of type I IFN in several tissues (lung, liver, spleen). This phenomenon is now known to occur through recognition of dsRNA by TLR3 or the above cited RNA helicases. However, PKR deficient mouse embryonic fibroblasts exhibited a diminished response to IFNγ and polyrI-polyrC for induction genes responsive to the Interferon Regulatory Factor 1 (IRF-1) and the Nuclear Factor kappa-light-chain-enhancer of activated B cells (NF-κB), which presented evidence for a participation of PKR in these signaling pathways [81]. It was later shown that PKR can activate the NF-κB pathway by interacting in a complex with the IKKβ kinase (Inhibitor of nuclear factor kappa-B kinase subunit beta kinase). In this process, the catalytic activity of PKR may favor activation of NF-κB [82] but is not absolutely required [83,84]. An attracting possibility is that the formation of the complex involves TNFR-associated factor (TRAF) proteins, which serve as a link between the N terminus of PKR and the TRAF family member-associated NF-κB activator (TANK)/ NF-kB Essential Modulator (NEMO) complex that recruits IKKβ [10,16]. 

#### 4.3.3. PKR and MAPKinase Pathway

The Mitogen-Activated Protein Kinases (MAPKs) are serine/threonine kinases which act in cascade in response to external or internal stimuli and regulate multiple cellular pathways. Schematically, in response to a stimulus, there is first activation of a member of the MAPKKK subset, which phosphorylates a member of the next element in the cascade, a MAPKK, which activates in turn a MAPK. The most extensively studied MAPK pathways are those leading to activation of the MAPK ERK 1/2 in response to extracellular growth factors and to the activation of the p38 mitogen-activated protein kinase (p38 MAPK) and the c-Jun NH2-terminal kinases (JNK) in response to a stress [85]. Intracellular accumulation of dsRNA, such after transfection with polyrI-polyrC leads to activation of both p38 MAPK and JNK, as well as their upstream MAPKK (MKK3/MKK6 for p38 MAPK and MKK4/MKK7 for JNK), but not of ERK1/2 and its upstream kinase MEK1/2. JNK activation could result from the disappearance of a labile negative regulator due to an inhibition of translation following activation of both PKR and Ribonuclease Latent RNAse L [86]. The use of cells lacking both PKR and RNAse L alleles showed that the p38 MAPK pathway is still activated in response to polyrI-polyrC, indicating that a novel dsRNA-induced pathway leads to p38 MAPK activation [86]. In light of recent findings, this activation can most probably be attributed to the RIG-I/MDA5 pathway [87,88]. The relationship between PKR and activation of the p38 MAPK pathway is complex and still uncompletely understood. PKR was shown to interact with and activate MAPKK6 in a dsRNA-dependent manner, which leads to p38 MAPK activation. However, incubation of PKR deficient MEFs with polyrI-polyrC still lead to phosphorylation of p38, although to a lesser level than in PKR wt MEFs, indicating that PKR is involved, but not fully responsible for the modulation of p38 phosphorylation [89]. Infection of HeLa cells but not HeLa cells knocked down for the expression of PKR with a mutant Vaccinia virus lacking E3L leads to a PKR-dependent phosphorylation of p38 [90,91]. However, PKR deficient MEFs treated with TNF can respond with increased phosphorylation of p38 MAPK [92]. This indicates that modulation of the p38 MAPK pathway by PKR depends not only on the stimulus applied to the cells (dsRNA, virus, tumor necrosis factor (TNF)) but also on the level of PKR expression in the cells. 

#### 4.3.4. PKR and the Insulin Pathway

**Figure 3:**
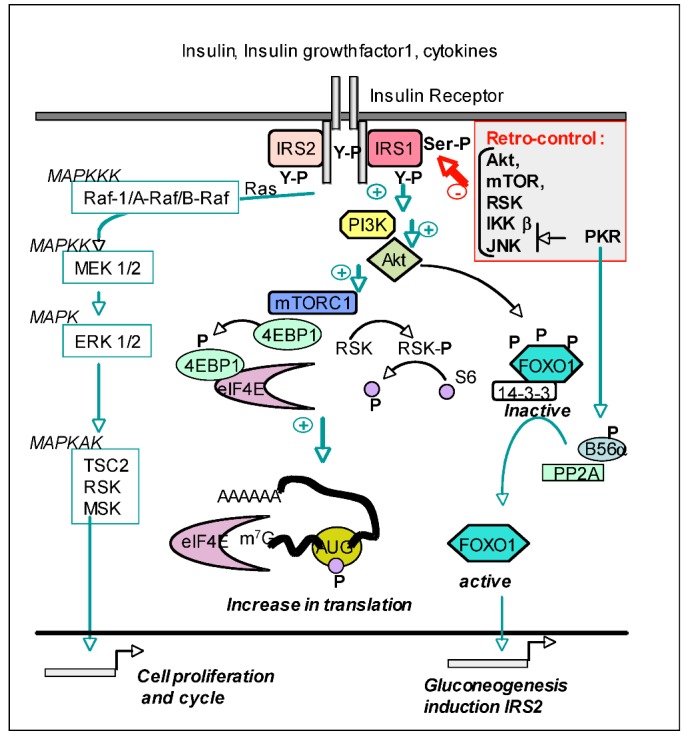
PKR and the Insulin signaling pathway. After binding of the insulin receptor to its different ligands, its intracytosolic chains autophosphorylate on tyrosine residues and phosphorylate the two substrates IRS1 and IRS2 on tyrosine residues to recruit the SH2‑containing Phosphoinositol-3 kinase (PI3K). Lipid products generated by PI3K bind and activate Akt, which in turn activates mTORC1. The latter phosphorylates 4EBP1 and RSK (Ribosomal S6 kinase). As a result, 4EBP1 dissociates from the cap-binding eIF4E protein and RSK phosphorylates the S6 ribosomal protein. The outcome of this is an increase in translation. Activation of the insulin receptor triggers also the Mitogen Activated Protein Kinase (MAPK) cascade (reviewed in [85]) leading to the transcription of factors required for cell proliferation and cycle. Akt also negatively regulates the Forkhead BoxO transcription factor FOXO1 by phosphorylation. This maintains FOXO1 in the cytosol in a complex with the 14-3-3 protein and prevents gluconeogenesis or transcription of IRS2 when not needed. Retro-control of the insulin pathway occurs through phosphorylation of IRS1 on serine residues by Akt, mTOR, RSK and by PKR, the latter acting through the intermediate IKKβ or JNK kinases. This prevents its interaction with the insulin receptor. However, PKR can also positively activate the insulin signaling by triggering FOXO1 dephosphorylation through the phosphorylation of B56α, the regulatory subunit of the protein phosphatase PP2A.

The insulin signaling pathway plays an essential role in relationship with glucose and lipid metabolism and is subject to a complex regulation. After binding to its ligand, the insulin receptor is phosphorylated on tyrosine residues and can recruit its two substrates: Insulin Receptor Substrate 1 and 2 (IRS1 and IRS2), which are themselves phosphorylated on tyrosine residues and serve as adapters to initiate several transduction cascades, through the kinases: Phosphoinositol-3 Kinase (PI3K), Akt (mouse Ak strain with thymic lymphoma; also known as Protein Kinase B (PKB)), and mammalian Target of Rapamycin (mTOR). Akt activates by phosphorylation and upon binding of lipid products generated through the action of PI3K. It leads to activation of the complex mTOR1 (mTORC1), which, among other functions, triggers an increase in translation through phosphorylation of eukaryotic translation initiation factor 4E-binding protein (4EBP1) which dissociates from the cap-binding initiation factor eIF4E. It also triggers the phosphorylation of the S6 kinase, which leads to phosphorylation of the S6 ribosomal protein and its binding with the translation machinery on 43S RNA. Retro-control of the insulin pathway occurs through inhibition of the activity of both Akt and mTOR by the phosphoinositide phosphatase and TENsin homolog (PTEN) and also through the ability of mTOR, S6K, IKKβ and JNK kinase to phosphorylate IRS1 on serine residues. This prevents its interaction with the intracytosolic domain of the insulin receptor [93]. Interestingly, PKR can also modulate both positively and negatively the insulin signaling pathway. On one hand, PKR can activate the transcription factor FoxO1 through the phosphorylation of B56α, the regulatory subunit of the protein phosphatase PP2a. Once activated by phosphorylated B56α, PP2a dephosphorylates and activates FoxO1. Among its different activities, FoxO1 stimulates transcription of IRS2 and then sustains the insulin signaling pathway. On the other hand, PKR negatively regulates the insulin signaling pathway by triggering serine phosphorylation of IRS1, either directly and/or through an intermediate kinase, such as IKKβ or JNK [94,95] (Figure 3). 

## 5. PKR and Its Role in HCV Infection

### 5.1. Interaction of HCV with PKR

#### 5.1.1. PKR and HCV IRES

**Figure 4:**
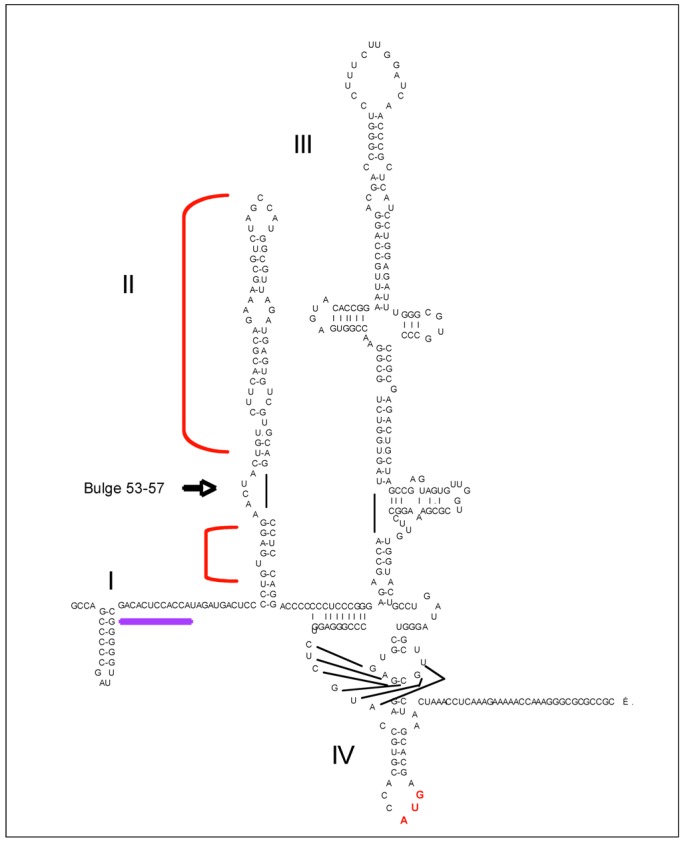
PKR and the HCV IRES. Secondary structure of the Internal Ribosomal Entry site (IRES), located within the first 388 nucleotides of HCV RNA. The four stem-loop domains of the IRES are numbered in Roman numerals (I, II, III and IV). The binding site for miR122 is underlined (purple). The brackets (in red), located on each side of a 53–57 bulge (arrow) indicate the protected areas from ribonuclease or 2' hydroxyl attack when PKR is present.

The HCV single-stranded positive-sense RNA of approximately 9,600 bp presents a unique open reading frame encoding the viral polyprotein which is flanked with two highly structured 5' and 3' ends. The 5' end is devoid of a cap structure and contains an IRES with four stem-loop domains, numbered I to IV. Of note, immediately after domain I, there is a binding site for microRNA (miR)122, which has a strong positive effect on HCV translation [96] The loops II, III and IV are involved in the initiation of translation through their IRES structure. Binding of domain II to the ribosome 43S triggers a conformational change that helps the junction with the 60S subunit and the formation of the 80S ribosome. Domain III helps positionning the HCV start codon, located in domain IV, at the ribosome start site [97] (Figure 4). The HCV IRES has the possibility to activate PKR, in spite of its complex structure and the fact that longer base-pair stretches (at least 33 bp) are usually required to activate PKR [98]. Indeed, *in vitro* binding experiments between purified PKR protein and IRES showed that PKR can be activated upon binding to both domain II and domain III-IV and that domain II is the strongest activator [37,97]. Ribonuclease footprinting and in-line cleavage protection from 2'hydroxyl-attack of domain II in complex with the N terminal domain containing the two DRBDs of PKR (1–184) showed that PKR binds above and below the AACUA bulge (nt 53–57) of this domain. Further footprinting assays using domain II mutants showed that this bulge is also required and may be involved in the correct positioning of PKR [97]. However, there is currently no direct demonstration that HCV IRES is responsible for activation of PKR *in vivo*. PKR activation has been reported in HCV subgenomic replicon cell lines during HCV replication, as detected by using antibodies directed either against phosphorylated PKR [99] or against phosphorylated eIF2α [99,100]. PKR has also been shown to be activated upon infection of different Huh7 cell lines with the Japanese Fulminant Hepatitis-1 (JFH1) HCV strain [38,101,102,103]. Immunoprecipitation of PKR from JFH1‑infected cells followed by RTqPCR of HCV RNA demonstrated that PKR can interact with HCV RNA early in infection but without sign of PKR activation as a kinase [103]. Therefore, the ability of HCV IRES to activate PKR *in vivo* should be further characterized.

#### 5.1.2. PKR and Core Protein

**Figure 5:**
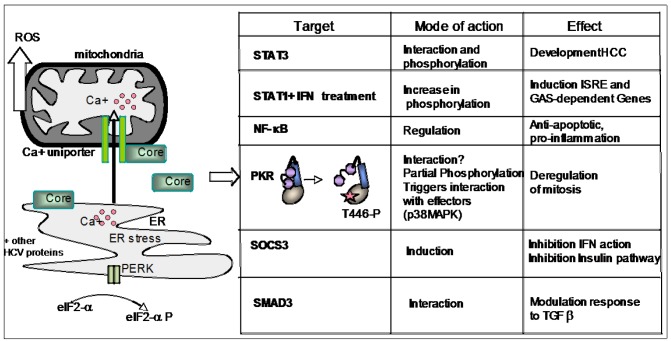
PKR and the HCV core protein. After translation from the HCV RNA, the HCV proteins, including the core protein, interact with the membrane of the endoplasmic reticulum (ER). Accumulation of the core protein at the ER triggers an ER stress which can lead to a PERK-mediated eIF2α phosphorylation and also a release of calcium (Ca+). The core protein binds also to the outer membrane of mitochondria where it stimulates the Calcium uniporter, thus allowing calcium uptake and generation of Reactive Oxygen Species (ROS). The core protein interacts with multiple cellular proteins and affects a number of signaling pathways. Some of its targets are shown in the left table, with indications of its mode of action and effect on the cells. The core protein interacts with PKR to trigger its partial phosphorylation (on threonine 446) which may change the conformation of PKR and favor its interaction with other partners such as p38MAPK.

The HCV core protein is the first protein to be translated from the HCV polyprotein as a 191 amino acid form. It is then processed to a 189 aa mature form after cleavage of a signal peptide, at the endoplasmic reticulum (ER). The core protein plays an essential role in the virus assembly process. In addition, it is involved in multiple transduction pathways and its deregulated expression is mostly regarded as leading to HCV-related pathogenesis, such as steatosis and hepatocellular carcinoma [104,105]. From its ability to reside at the ER, similarly to the other HCV proteins, the core protein can trigger an ER stress with subsequent release of calcium. Furthermore, the core protein can also bind the outer mitochondrial membrane and stimulate the mitochondrial calcium uniporter, thus favoring calcium uptake in mitochondria and subsequent generation of oxydative stress [106]. In addition, the core protein can interact or activate different proteins involved in multiple signaling pathways, such as NF-κB [107,108], Signal Transducer and Activator of Transcription (STAT) 1 [109,110] and 3 [111,112], Suppressor Of Cytokine Signaling 3 (SOCS3) [113,114] and SMAD family member 3 (SMAD3) [115,116] (Figure 5). PKR belongs also to the list of proteins associated to the action of the core protein. In this case, eIF2α phosphorylation, a convenient read-out for PKR activation, has to be considered with caution since it can also be triggered through PERK activation in response to core‑mediated ER stress. Introduction of the core protein in HepG2 cells and in MEFs deficient for PKR as well as use of different PKR catalytically mutants showed that the core protein was able to trigger an increase in the G2/M phase and cell cycle deregulation, in a manner that involves the phosphorylation of PKR only on its residue T446 with no subsequent phosphorylation of its residue T451 and therefore does not lead to PKR activation [117]. The cell cycle deregulation would act through a p38 MAPK/PKR-dependent pathway due to a strong interaction of PKR with p38 MAPK in HCV core-expressing cells [118]. In this mechanism, an interaction between PKR and core could not be established [117]. However, another report showed that the core protein can interact with the N terminus of PKR through its first 58 amino acids [119]. Formal demonstration of a PKR/core interaction should be performed using HCV propagated in cell culture (HCVcc) or using liver biopsies in HCV-infected patients. In particular, it should be interesting to determine whether and where core and PKR colocalize in HCV-infected cells, as both of them, which are essentially in the cytosol, can also be found in the nucleus [21,120,121]. As mentioned above, PKR can negatively modulate the insulin pathway. The HCV 1b core protein interferes also with the insulin pathway, through induction of SOCS3, a member of the family of the SOCS proteins. In addition to its best known ability to interfere with the IFN-responsive pathway [113], SOCS3 can trigger ubiquitination and proteasome-mediated degradation of IRS1 and IRS2, thus blocking activation of PI3-kinase/Akt/6-phosphofructo-2 kinase and entry of glucose into the cells [114]. Regardless of their ability to interact or not, the action of PKR and core may thus converge to deregulate important signaling pathways such as that of the insulin pathway. This would account, at least in part, for the insulin resistance phenotype associated to HCV chronic infection.

#### 5.1.3. PKR and NS5A

**Figure 6:**
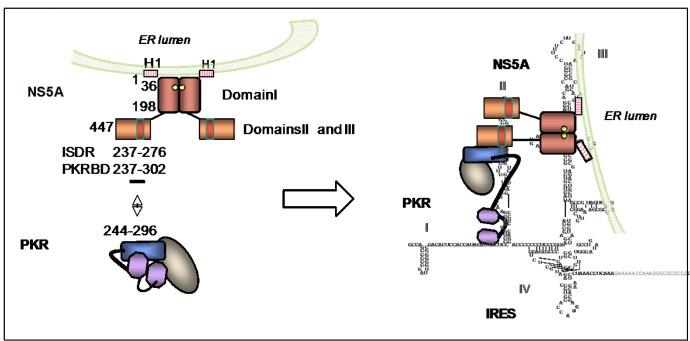
PKR and the HCV NS5A protein. The 447 aa NS5A protein associates as a dimer and is anchored in plane to the cytosolic part of the ER through its amphipathic helix (H1) present in its domain I. NS5A interacts with HCV RNA through its domain I. Its domains II and III are flexible and able to interact with different proteins. The ISDR (237–276) and PKR binding domain (PKRBD: 237–302) are located in domain II (red bar). NS5A can interact with the 244–296 region of PKR corresponding to the N lobe of the catalytic domain of PKR. This interaction may benefit from the binding of NS5A and PKR to the domains III and II of HCV RNA, respectively.

Historically, NS5A was the first HCV protein to have been linked to a control of antiviral cellular defense by this virus. A strong correlation between the sensitivity or resistance of patients to IFN treatment and the number of mutations in the 237–276 amino-acid region of this protein, thus referred to as Interferon Sensitive Determinant Region (ISDR), was noted in patients chronically infected with HCV of genotype 1b [122]. This correlation was, however, challenged with data from other studies performed on European patients infected with HCV of genotype 1a or 1b [123,124]. A recent comparison of sequence from full HCV genomes before IFN/Ribavirin (RBV) therapy of patients infected with genotype 1a or 1b showed that the HCV sequences from responders are more diverse than those of non-responders and that the NS5A sequence, but also core and NS2, present differences [125]. Following the initial link between the ISDR and response to IFN treatment, PKR was proposed as a possible target for NS5A. By yeast two-hybrid technology, an interaction was found between the dimerization region 244–296 of PKR and the 237–302 region of NS5A, containing its ISDR and the following 26 residues. This interaction was confirmed after cotransfection of PKR and NS5A constructs in Cos-1 cells [126,127] but not when using a human cell line inducibly expressing the structural and nonstructural proteins derived from the prototype HCV-H strain (genotype 1a) [128]. However, NS5A was able to inhibit PKR activation and subsequent eIF2α phosphorylation in the context of infection with a Vaccinia virus in which the E3L protein, a powerful PKR inhibitor, was replaced by NS5A [129]. The interplay between PKR and NS5A still requires to be studied in the context of HCV infection, in view of the specific subcellular localization of NS5A, its regulation by multiple kinases and its important role in the replication and production of HCV [130,131]. Structural analysis of this 447 amino acid protein revealed that its domain I is highly structured in amphipathic helix and that its domains II and III are flexible. NS5A associates as a dimer and is anchored in the ER membrane via its N terminal domain I [132]. As such, NS5A can bind G/U rich regions of HCV RNA through a basic pocket turned towards the cytosol, leaving its domains II and III free to bind cellular proteins [133] (Figure 6). There is currently no direct demonstration that PKR is inhibited by NS5A during HCV infection, but it could occur due to the better affinity of NS5A for the stem-loop III-IV than for the stem-loop II of the HCV IRES RNA structure, while stem-loop II is a better PKR activator than the other IRES domains. The domain II of HCV IRES is involved both in replication and translation, whereas domain III-IV is required only for translation. It has been proposed that, at the beginning of infection, NS5A remains associated with domain III-IV, thus leaving domain II available for PKR activation and subsequent eIF2α-mediated inhibition of the translation of cellular mRNAs. NS5A then accumulates to eventually interact with PKR, due to their proximity on the two IRES domains, thus leading to subsequent inhibition of PKR. Although direct experimental evidence is still lacking, this would explain how the accumulation of mutations in the ISDR regions may abrogate the NS5A/PKR interaction [97]. 

#### 5.1.4. PKR and E2

Some sequence identity was noticed between the E2 envelope of HCV genotype 1a or 1b and with PKR and eIF2α. This was referred to as the PePHD region (PKR-elF2-alpha phosphorylation Homology Domain) [134]. In this study, E2 binds to and inhibits PKR *in vitro* and after expression in yeast and enhances translation of cellular proteins after expression by transient transfection. The region of PKR homologous to that of E2 lies within its flexible linker between the two dsRBDs, which can be phosphorylated on its serine 83 and threonines 88, 89 and is considered to favor the wrapping of PKR around the dsRNA. This mimicry of the autophosphorylation site by HCV E2 may contribute to PKR inhibition via a pseudosubstrate mechanism [135]. Although E2 weakly affects PKR activity *in vitro*, it may have an impact on PKR expression *in vivo*. However, E2 is essentially accumulating in the lumen of the endoplasmic reticulum (ER), whereas PKR resides in the cytosol. It has been proposed that an E2/PKR interaction may result from the presence of some unglycosylated 38 kDa E2 species that localize in the cytosol and are rapidly degraded through the proteasome unless they interact with PKR [136]. E2 was also shown to inhibit the PKR-related kinase, PERK, by preventing its access to eIF2α in this case [137].

Any correlation between variations in the sequence of HCV genome and the response or non‑response of patients to treatment is of high clinical interest to predict the outcome of therapy. However, a large number of analyses of variations in the PePHD region as well as in the ISDR regions, in patients infected with HCV only or coinfected HCV/HIV, highlighted only a correlation between the ISDR region and the positive response to treatment [138]. Thus, the importance of the PePHD region still remains to be determined. Whatever the case, the HCV proteins E2 and NS5A are the major viral proteins with potential determinants of genotype-specific clinical outcome, as shown by a recent phylogenetic analysis with all available full-length HCV genomic sequences (n = 345) showing the relative evolutionary ages of the major HCV genotypes [139]. 

### 5.2. HCV and the Innate Immune Response

#### 5.2.1. HCV and Activation of the Pathogen Recognition Receptors

**Figure 7:**
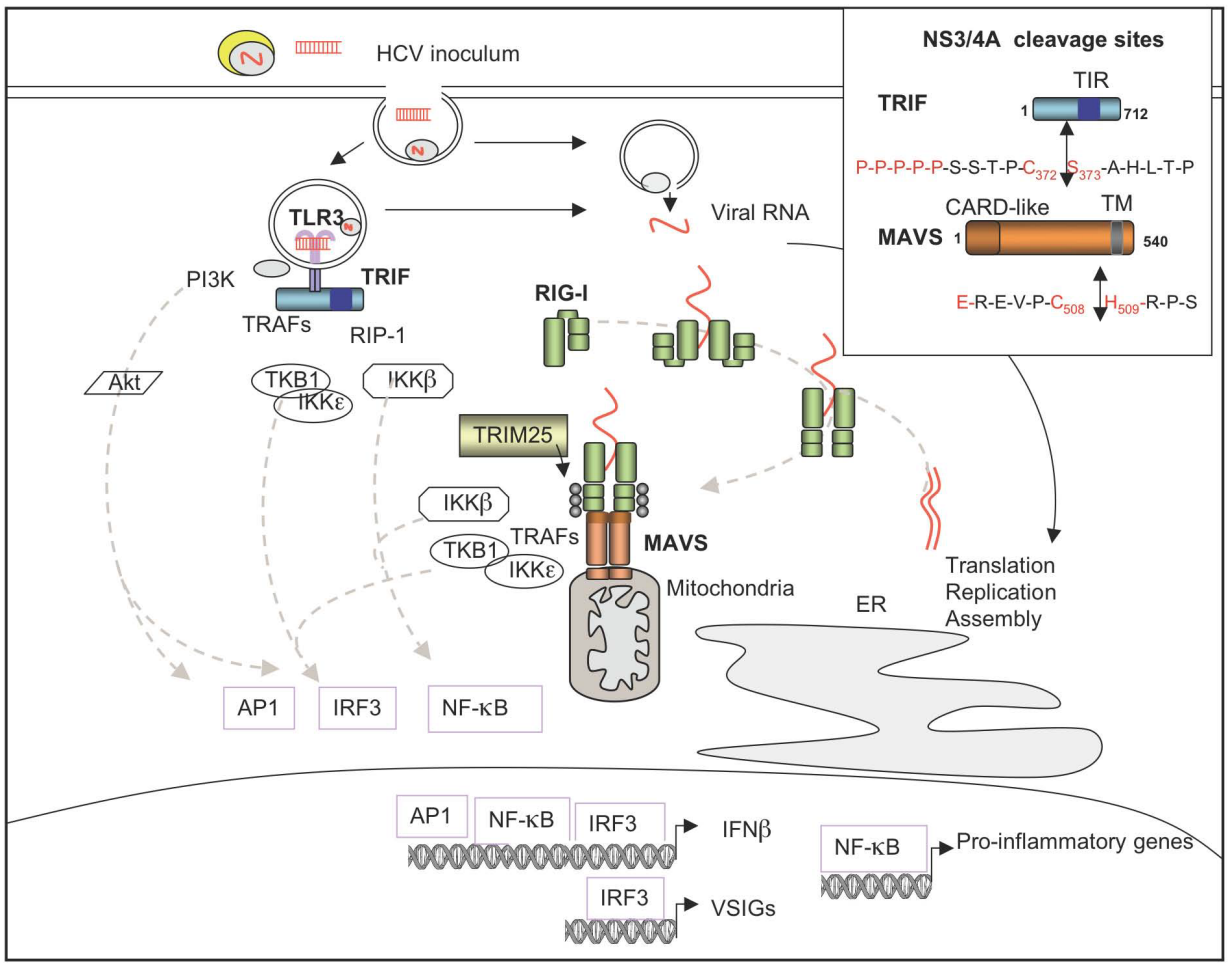
HCV and induction of the innate immune response. HCV activates the innate immune response through the endosomal TLR3 via dsRNA structures (derived from apoptotic of necrotic infected cells) present in the inoculum and through the intracytosolic RNA helicase RIG-I after entry of the viral RNA into the cytosol. Activation through TLR7 is not represented here. Binding of RIG-I to viral dsRNA structures triggers a change in its conformation and its dimerization/multimerization. RIG-I becomes ubiquitined (grey circles) by the E3 ligase TRIM25, which allows its interaction with the mitochondria-bound protein MAVS [157] (MAVS is also known as Cardif [158], VISA [159] and IPS-1 [160]). MAVS recruits different adapters such as members of the TRAF family and the downstream kinases IKKβ, TBK1 and IKKε which activate the transcription factors NF-κB and IRF3, respectively. In addition, the MAVS pathway can also lead to activation of AP-1 (ATF-2/C-jun) (not represented). Upon binding to dsRNA, TLR3 recruits the intracytosolic adapter TRIF, which itself recruits members of the TRAF family, the adapter RIP-1 (Receptor-Interacting Protein 1) and the NF-κB- and IRF3‑activating kinases. In addition, the TLR3 pathway triggers induction of AP-1 through PI3K/Akt. Combination of the three transcription factors is necessary for efficient induction of IFNβ, while IRF3 alone is sufficient for induction of a series of genes, referred to as VISGs (Virus Stress Inducible Genes), which will be later on responsive to IFN. NF-κB stimulates the transcription of pro-inflammatory genes. Box: The HCV NS3/4A protease has the capacity to abrogate induction of the innate immune response through the cleavage of TRIF, close to its Toll-like/IL-1 Receptor (TIR)-domain and of MAVS, close to its transmembrane domain. The sequence of the cleavage sites is shown. CARD stands for Caspase Recognition Domain.

An elevation of the T CD4^+^ et CD8^+^ response occurs significantly in some of the HCV-infected individuals who are able to spontaneously eliminate this virus (15%–30% of cases), which indicates that the host has the ability to mount an immune response against this pathogen [140,141]. Indeed, HCV has an intrinsic ability to induce IFN and the innate immune response, via some of the dedicated Pathogen Recognition Receptors (PRR), such as the DExD/H-box Helicase RIG-I and the Toll-like Receptors TLR3 and TLR7 (reviewed in [142]) (Figure 7). Interaction between RIG-I and HCV RNA occurs essentially between the 3' UTR region of the genomic RNA, with a preference for the U/C rich sequences [143,144,145]. The HCV 3' UTR has a better affinity for RIG-I than other 3' UTR of other close RNA viruses such as the flavivirus Dengue virus, West Nile virus or Yellow Fever Virus [146]. RIG-I can form multimers in a cooperative manner on dsRNA, implying that the efficacy of the activation signal may increase during the replicative phase of the virus [147]. HCV can also trigger innate immunity through TLR3 but the exact motif used as PAMP (Pathogen Associated Molecular Pattern) in its RNA structure has not been identified yet [148]. TLR3-overexpressing hepatocytic cells present a better ability to restrict HCV replication than control cells, it has been proposed that TLR3 activation occurs upon contact with dsRNA HCV structures such as replicative forms that would be accessible after phagocytosis of apoptotic or necrotic HCV-infected cells. This would then lead to the production of IFN, which can account for the observed activation of dendritic cells and of T cells and NK cells after HCV infection [149]. In addition to RIG-I and TLR3, TLR7 has also been recognized as a PRR for HCV. Activation was found to involve essentially the poly(U) rich region located at the 3' UTR of HCV genomic RNA [150]. It has been reported that HCV RNA can be transferred from HCV-infected hepatocytes to plasmacytoid dendritic cells (pDCs) through a novel mechanism that remains to be defined but requires cell-to-cell contact. This leads to induction of IFNα from the pDCs in a TLR7‑dependent manner [151].

#### 5.2.2. Control of IFN Induction by HCV NS3/4A

The HCV NS3/4A protease plays an important role in the control of the innate immune response by HCV. NS3/4A negatively regulates the TLR3 and RIG-I pathways, through cleavage of their respective adapters: The Toll-like/IL-1 Receptor (TIR)-domain-containing adapter-inducing interferon-β (TRIF) and the Mitochondrial Antiviral Signaling protein (MAVS) (Figure 7). NS3/4A cleaves TRIF between its residues Cys-372 and Ser-373, located upstream of the TIR. This property has been initially demonstrated *in vitro* [152], then after transient transfection [153]. Degradation of TRIF has also been observed three days after infection of the Huh7.5 cells by HCVcc and its expression was restored when cells are treated with a specific inhibitor of the NS3/4A protease activity, thus demonstrating the participation of NS3/4A in this process [148]. Inhibition of the TLR3/TRIF could help HCV to control the immune response triggered by dsRNA structures released together with cell debris from HCV-infected cells in the extracellular space. Trapping of these dsRNAs by TLR3 would reduce their ability to activate circulating dendritic cells for the production of IFN and cytokines while their ability to trigger IFN induction would be blocked within the infected hepatocytes through NS3/4A. However, it is possible that some hepatocytes may respond well to extra-cellular dsRNA to induce ISGs and IFN, before or at the beginning of the infection. In regard with the RIG-I pathway, NS3/4A cleaves MAVS after its cysteine 508 residue, close to its 514–535 transmembrane domain. This leads to a disruption of MAVS from the outer membrane of mitochondria, where the majority of MAVS resides [154]. As a consequence, downstream partners of MAVS that are engaged in the activation of Interferon Regulatory Factor 3 (IRF3) (Figure 7) are also delocalized to the cytosol and cease their activity [155]. MAVS cleavage has been confirmed *in vivo* in 48% of liver biopsies analyzed in 129 chronically infected patients and was more extensive in patients with a high HCV viral load [156]. The NS3/4A-mediated control of MAVS plays therefore an important role in the control of IFN induction by HCV. 

#### 5.2.3. HCV and Biomarkers Linked to the Immune Response

In spite of its ability to be recognized by PRR, HCV infection becomes a chronic disease in 60%–80% of the infected individuals. Novel biomarkers of the infection are constantly under active search to continue the understanding of the interaction between HCV and its host, its ability to escape the immune response and to adapt the therapy. A recent screening approach using overexpression of 380 human ISGs showed that HCV replication was the most strongly inhibited by those that are continuously fueling the IFN system, such as the DExD/H-box Helicases RIG-I and/or MDA5 and the transcription factors IRF1 and IRF7 (Interferon Regulatory Factor) [161]. Yet, paradoxically, HCV‑chronically infected patients that will respond the least to the IFN/Ribavirin therapy have long been reported to present a high intra-hepatic expression of ISGs in the absence or with low production of type I IFN [162,163,164]. This is considered as a strong negative predictive biomarker of the response to therapy. One important goal is to determine which of these ISGs are compromising the efficacy of the immune response. One of these ISGs is the pro-inflammatory chemokine C-X-C motif chemokine 10 (CXCL10) or Interferon Inducible Protein 10 (IP10), which recruits activated T cells to the liver in combination with other chemokines or effectors [165]. Only low levels of IP10 prior to treatment is predictive of a Rapid Virological Response (RVR) and a Sustained Virological Response (SVR) [166]. It is possible that IP10 compromises their ability to clear HCV due to its circulation as a truncated antagonist form when highly expressed in HCV-infected patients [167]. The Ubiquitin Specific Peptidase 18/Ubiquitin Binding Protein 43 (USP18/UBP43) is another ISG that can compromise the immune response by interfering with the response to IFN, although a direct relation between the function of this ISG and HCV therapy has not been provided yet. In addition to its isopeptidase activity, USP18/UBP43 can desensitize the cells to treatment by IFNα because of its ability to bind to the intracytosolic domain of the Interferon-α/β Receptor 2 (IFNAR2) chain of the Type I IFN receptor [168,169]. Interestingly, the signaling response to IFNβ, another Type I IFN, or to IFNλ, a Type III IFN, is not affected because IFNβ has a better interaction with IFNAR2 than IFNα and because the signal triggered by IFNλ does not involve IFNAR2 [170,171]. Indeed, IFN-λ1 was found efficient in the treatment of patients who were refractory to treatment with IFNα [172]. These data highlight the increasing importance of IFNλ. In regard with this, the fact that IFNλ belongs also to the family of ISGs, in addition to be an IFN, places this molecule in the interesting position to regulate the innate immune response through induction of ISGs and by controlling the response to type I IFN. IFNλ1 is also known as IL29. Two other members of this group of type III IFN are IL28A (IFNλ2) and IL28B (IFNλ3). A search for an association between Single Nucleotide Polymorphism (SNPs) and disease led to the discovery of an association between SNPs in the IL28B gene and HCV clearance [173,174,175]. Although the mechanism involved is currently not known, the analysis of IL28B polymorphism, in combination with relevant biomarkers, such as IP10, provides a strong predictive response to therapy [176]. 

#### 5.2.4. PKR and ISG15, Two ISGs as Pro-HCV Agents

**Figure 8:**
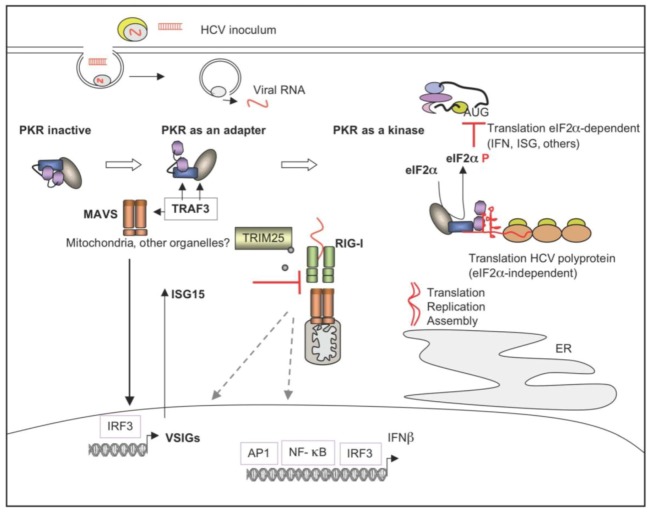
PKR, ISG15 as modulators of IFN induction during HCV infection. HCV triggers associations of PKR with TRAF3 and MAVS early in infection. This involves the first DRBD of PKR but leaves PKR inactive as a kinase. The PKR/MAVS association leads to the activation of IRF3 and induction of VSIGs. Among these, ISG15 prevents the TRIM25-mediated ubiquitination of RIG-I and its association with MAVS, thus inhibiting the IFN induction pathway. Later in infection, PKR is activated as a kinase, phosphorylates eIF2α, with subsequent transient inhibition of the general translation including IFN and IFN-Stimulated Genes (ISGs) while the translation process of the HCV polyprotein proceeds unabated since it is eIF2α-independent.

Two different approaches, in our group [38] and the Chisari group [102], studied the relationship between the ability of HCV to regulate IFN induction or the response of HCV-infected cells to IFN, led to the observation that HCV infection triggers phosphorylation of PKR. This leads to subsequent phosphorylation of eIF2α and attenuation of protein synthesis, including that of IFN [38] and of the ISGs [102]. This situation is therefore in favor of the translation of the viral proteins, since translation from the HCV IRES is independent of eIF2α phosphorylation [177,178]. Both studies differed in their use of JFH1-permissive cells, such as use of the Huh7.25/CD81clone [179] in the Arnaud study [38] or Huh7 cells [180] in the Garaigorta study [102] but they similarly concluded that the suppression or inhibition of PKR leads to the inhibition of HCV yields provided that the cells have a robust IFN induction pathway [38] (Figure 8) or are treated with IFN [102]. These findings led to the notions that HCV can use PKR as a proviral agent. An important consequence of this is that the antiviral action of IFN against HCV may benefit from the absence of PKR. It is interesting to recall here that the HCV NS5A or E2 proteins were previously noted as PKR inhibitors. Since PKR acts as a pro-HCV agent, this is raising the question why HCV is using two of its proteins to counteract the action of PKR.

The Huh7.25/CD81 cells are convenient to study the IFN induction pathway in response to HCV infection because they express a functional RIG-I, in contrast to the widely used HCV-permissive Huh7.5 cells, where a T55I substitution in the first CARD domain of RIG-I prevents its ubiquitination by the E3 ligase TRIM25/EFP (Tripartite Motif containing protein 25/Estrogen-responsive ring Finger Protein) and hence its association with MAVS [143]. Intriguingly, we observed that a correct activation of the IFN induction pathway in these cells in response to HCV required overexpression of the TRIM25/EFP. This E3 ligase mediates not only the conjugation of ubiquitin but also that of ISG15, a process referred to as ISGylation. We have examined the participation of ISG15 in the IFN induction pathway in response to HCV infection. ISG15 is a di-ubiquitin-like protein also known as ubiquitin cross reactive protein (UCRP) [181,182]. It can also be induced rapidly by IRF3 early in viral infection [183]. Importantly, ISG15 is highly expressed in the liver of HCV chronically infected patients and considered as negative predictive biomarker of the ability of the patients to respond to therapy [162,163,164]. Indeed, a pro-HCV role for ISG15 has been reported and its action was suggested to occur at the level of viral protein synthesis or replication of the virus [184,185]. In experiments using cotransfection with ISG15 and the ISG15-conjugating enzymes, ISG15 and an ISGylation process were shown to negatively regulate RIG-I and thus to inhibit the signaling process leading to IFN induction [186]. Depletion of ISG15 was sufficient to restore ubiquitination of RIG-I in response to HCV infection, as well as to increase the transcription of IFN, while depletion of the ISG15-specific E1 ligase UbE1L abrogates the RIG-I induction pathway [103]. The mechanism used by ISG15 to control RIG-I ubiquitination has not been elucidated and may involve a competition between ISG15 and ubiquitin for their common E2 enzyme, UbcH8. Another possibility is that the E3 ligase TRIM25 is conjugated by ISG15, which would prevent its ability to ubiquitinate RIG-I [187] (Figure 8). Alternatively, the major ISG15 E3 ligase HERC5 (HECT and RLD domain containing E3 ubiquitin protein ligase 5) may also compete with TRIM25 to recruit UbcH8-ISG15. In each of these hypotheses, depletion of ISG15 restores the process of RIG-I ubiquitination. We have also noticed that PKR activation after HCV infection was strongly inhibited in the absence of ISG15 while it was increased by its overexpression [103]. Thus, both PKR and ISG15 can act as pro-HCV effectors. 

#### 5.2.5. PKR and Modulation of Pathways Related to Innate Immunity

While analyzing the regulation of IFN induction pathway by ISG15 in JFH1-infected Huh7.25/CD81 cells, we noticed that the expression of ISG15, as well as ISG56, another IRF3‑responsive ISG, increased in response to JFH1 infection in the Huh7.25/CD81 cells. This was also observed in the Huh7.5 cells, Huh7 cells and human primary hepatocytes [103]. Interestingly, induction of ISG15 in the Hu7.5 cells was also reported in response to J6/JFH-1, another HCVcc [185]. This induction requires an early event that occurs ahead of the RIG-I/MAVS pathway and involves PKR as an adapter protein in a complex with MAVS and TNF Receptor-Associated Factor 3 (TRAF3), with no requirement of its kinase function. Rapid induction of ISG15 through this pathway has a dual function: (i) it controls the RIG-I pathway by interfering with RIG-I ubiquitination, hence limiting IFN induction, and (ii) it maintains PKR phosphorylation later in infection [103] (Figure 8). The mechanism that leads to the recruitment of PKR and allows its participation in signaling pathways related to the control of innate immunity in response to HCV infection is still not clear, but examples in other models of viral infection could help its understanding. For instance, PKR is also involved in pathways leading to IFN induction during infection with bluetongue virus, which has a dsRNA genome. In this case, induction of IFN in pDC by this virus, does not involve TLR7 as expected but occurs through Myeloid Differentiation primary response gene 88 (Myd88), PKR and JNK [188]. PKR can be engaged in several complexes by protein-protein interaction through its N terminus domain, and its TRAF-binding motifs. In addition, PKR has recently been shown to interact with the actin-binding gelsolin, regardless of its kinase activity and to alter the ability of human rhinovirus-16 (HRV-16) and HSV-1 to enter cells [189].

#### 5.2.6. A Role for Pharmalogical Inhibitors of PKR?

Because PKR belongs to the arsenal of IFN-induced proteins and because of the accumulation of evidence that several virus-encoded products can interfere with the action of PKR or hijack PKR for their own profit, this protein is often considered essentially as a major actor in the antiviral action of IFN. Yet, PKR deficient mice can resist a number of viral infections, do not produce spontaneous tumors, are more suitable to resist stress and show strong cognitive network. Indeed, PKR is recognized for its negative effect on neurodegenerative disorders such as Huntington’s, Parkinson’s and Alzheimer’s diseases [190]. As shown above, PKR can be entangled in several signaling pathways leading to the activation of NF-κB, MAPKs, regulation of the insulin pathway and regulation of the IFN induction pathway itself. These data may help to consider developing pharmacological inhibitors of PKR either alone or in combination with IFN or direct antiviral agents as a reliable therapeutic approach to limit stress-induced damages or limit some viral infections. The first DRBD of PKR is essential for its activation and its Nuclear Magnetic Resonance (NMR) structure has been established [5,191]. PRI, a cell penetrating peptide containing the 21-aa peptide corresponding to an α‑helix of this DRBD was demonstrated to regulate PKR *in vivo* [192]. We have shown that PRI can prevent both the ability of PKR to act as a kinase to control translation of cellular proteins [38] and as an adapter to control the MAVS-related pathway of IFN induction in response to HCV infection [103]. Our data indicate that PKR inhibitors could be used in combination with IFN therapy to control HCV infection. PRI-mediated inhibition of PKR was also shown to attenuate the expression levels of the beta site amyloid precursor protein (APP)-cleaving enzyme 1 or BACE1 in response to oxydative stress in neuroblastoma cells [51], indicating that pharmacological inhibitors mimicking the PRI effect could be used in the treatment of neurodegenerative diseases. 

## 6. Conclusions

The dsRNA-activated protein kinase PKR participates in several cellular signaling pathways in addition to controlling protein translation through eIF2α phosphorylation. PKR was initially thought to be an essential actor in the mechanism of action of IFN, given the number of viral proteins that were reported to counteract its action. However, a role for PKR as a pro-viral agent is now emerging. This propriety can be particularly emphasized in the case of infection by the hepatitis C virus, where it acts both through its eIF2α kinase function and its ability to interact with components of the innate immune signaling pathway. PKR is now more often considered as a protein of stress. Its action can be either beneficial or harmful for the cell, depending on the nature of the stress, whether it is of viral origin or from other stimuli. Its action may also depend on its abundance in the cell. Pharmacological inhibitors of PKR have now to be considered seriously in order to be added to the arsenal of anti-stress agents.
